# Change the Qualis Criteria!

**DOI:** 10.1590/S1808-86942010000400001

**Published:** 2015-10-19

Since CAPES published its revised QUALIS criteria, the Brazilian Medical Association (AMB - Associação Médica Brasileira) has been organizing a series of meetings, held at its headquarters in São Paulo, Brazil, starting in August of 2009, in partnership with the Brazilian Association of Scientific Editors (ABEC - Associação Brasileira de Editores Científicos). These meetings led to the publication of an editorial entitled Classification of journals in the QUALIS System of CAPES – URGENT need of changing the criteria! The editorial was signed by 62 editors of scientific journals and published in full in all of them, and in countless others, primarily in the health sciences, demonstrating that Brazil's periodicals are more and more committed to discussing the problems they share in common.^1^

The scientific community remains concerned with the future prospects and direction of Brazilian periodicals.^2^, ^3^ The editors present at the meeting of the 18 of March were therefore in a position to evaluate the repercussions of the first Editorial, which had been discussed at scientific events and meetings all over Brazil. The meeting was attended by Dr. Lilian Caló, who is scientific communication and assessment coordinator for SciELO, and who presented a comparative study of the Brazilian periodicals indexed by SciELO according to two different criteria: the first criterion was the ISI/JCR impact factor, which only uses journals indexed by Thomson Reuters, and the second was an index composed by simple addition of the ISI/JCR and SciELO impact factors. The SciELO impact factor, which includes all the periodicals it indexes, significantly modifies the number of citations and, consequently, raises the Brazilian periodicals' impact factors. Dr. Caló's presentation illustrated this fact more clearly, showing the percentages gained by adopting the composite index. It is clear that combining indexes, or creating equivalencies or several alternatives can promote improved quality among Brazilian journals, raising their visibility and making international indexation more likely. It is also of concern that Brazilian researchers now prefer to publish their work in foreign journals rather than choosing domestic publications. They choose to do this because it improves the ratings of their postgraduate departments, earns them greater impact factors and increases their H index; all entirely and exclusively a result of the revised criteria adopted by CAPES. Achievement in increasing the visibility and quality of Brazilian scientific production should not be assessed on the basis of articles alone, but should also focus on improving our periodicals to the point at which they are recognized internationally.

Considering that the criteria have already been set for CAPES' current triennial assessment, the assembled editors decided to draft a second editorial containing a list of suggestions for the next assessment, to be sent to the administration at CAPES. The list of suggestions, which supplement those in the first editorial, is as follows:

– that the criteria used by CAPES to classify periodicals be revised, adopting the composite impact factor calculated by summing the ISI/JCR and SciELO impact factors;
– that a seat on the CAPES Scientific Committee be created for ABEC (the Brazilian Association of Scientific Editors) so that editor can be heard within the process;
– that CNPq be requested to create an “Editor's Scholarship” program to support scientific publishing and to be awarded to the editors of journals funded by CNPq. The objective of this project is to improve the quality of these journals by providing their editors with more time to dedicate to their editorial activities;

Additionally, the assembled editors decided to seek support for their criticisms and suggestions from the Brazilian Academy of Sciences (Academia Brasileira de Ciências), from FINEP and from Federal Deputy Eleuses Vieira de Paiva. At a later date the editors will request a detailed breakdown from CNPq of the criteria adopted for, and the results of, the distribution of the resources allocated through the Publishing Support Grants (Editais para Auxílio à Editoração). The editors intend to use these data to construct a database on the annual budgets of Brazilian periodicals, which will be useful for comparative analysis and mutual cooperation. Publication of these two editorials and promotion of discussion is one of our goals in seeking the recognition that Brazilian periodicals both need and deserve.

This editorial is signed by:

Adagmar Andriolo
Alfredo José Afonso Barbosa
Arnaldo José Hernandez
Aroldo F. Camargos
Benedito Barraviera
Bogdana Victoria Kadunc
Bruno Caramelli
Carlos Brites
Dejair Caitano do Nascimento
Domingo M. Braile
Dov Charles Goldenberg
Edmund Chada Baracat
Edson Marchiori
Eduardo de Paula Vieira
Eros Antônio de Almeida
Flávia Machado
Geraldo Pereira Jotz
Gianna Mastroianni Kirsztajn
Gilberto Camanho
Gustavo Gusso
Ivomar Gomes Duarte
Izelda Maria Carvalho Costa
João Ferreira de Mello Júnior
Joel Faintuch
José Antônio Baddini Martinez
José Eduardo Ferreira Manso
José Eulálio Cabral Filho
José Heverardo da Costa Montal
José Luiz Gomes do Amaral
José Luiz Martins
Jurandyr Moreira de Andrade
Leonardo Savassi
Luiz Augusto Casulari
Luiz Eugenio Garcez Leme
Luiz Felipe P. Moreira
Luiz Henrique Gebrim
Marcelo Madeira
Marcelo Riberto
Marcus Bastos
Mário Cícero Falcão
Mario J. da Conceição
Mauricio Rocha e Silva
Milton Artur Ruiz
Milton K. Shibata
Jornal Brasileiro de Patologia e Medicina Laboratorial
Jornal Brasileiro de Patologia e Medicina Laboratorial
Revista Brasileira de Medicina do Esporte
Revista Femina
Journal of Venomous Animals and Toxins including Tropical Diseases
Surgical & Cosmetic Dermatology da Soc. Brasileira de Dermatologia
Revista da Associação Médica Brasileira
Brazilian Journal of Infectious Diseases
Hansenologia Internationalis
Revista Brasileira de Cirurgia Cardiovascular
Revista Brasileira de Cirurgia Plástica
Revista da Associação Médica Brasileira
Revista Radiologia Brasileira
Revista Brasileira de Coloproctologia
Revista da Sociedade Brasileira de Clínica Médica
Revista Brasileira de Terapia Intensiva
Revista Brasileira de Cirurgia Cabeça e Pescoço
Jornal Brasileiro de Nefrologia
Revista Brasileira de Ortopedia
Medicina Família e Comunidade
Revista de Administração em Saúde
Anais Brasileiros de Dermatologia
Brazilian Journal of Otorhinolaryngology
Revista Brasileira de Nutrição Clínica
Jornal Brasileiro de Pneumologia
Revista do Colégio Brasileiro de Cirurgiões
Revista Brasileira de Saúde Materno Infantil
Revista da Associação Brasileira de Medicina de Tráfego
Revista da Associação Médica Brasileira
Archives of Pediatric Surgery
Revista Brasileira de Ginecologia e Obstetrícia
Revista Brasileira de Medicina de Família e Comunidade
Brasilia Médica
Geriatria & Gerontologia
Arquivos Brasileiros de Cardiologia
Revista Brasileira de Mastologia
Revista Brasileira de Mastologia
Revista Acta Fisiátrica
Jornal Brasileiro de Nefrologia
Revista Brasileira de Nutrição Clínica
Revista da Sociedade Brasileira de Anestesiologia
Revista Clinics
Revista Brasileira de Hematologia e Hemoterapia
Arquivos Brasileiros de Neurocirurgia
Mittermayer Barreto Santiago
Nelson Adami Andreollo
Nivaldo Alonso
Osvaldo Malafaia
Olavo Pires de Camargo
Paulo Manuel Pêgo Fernandes
Regina Helena Garcia Martins
Renato Soibelmann Procianoy
Ricardo César Pinto Antunes
Ricardo Fuller
Ricardo Guilherme Viebig
Ricardo Nitrini
Rogério Dedivitis
Ronaldo Damião
Rosângela Monteiro
Sergio Lianza
Sigmar de Mello Rode
Tarcisio E.P. Barros Filho
Wallace Chamon
Winston Bonetti Yoshida
Zuher Handar
Revista Brasileira de Reumatologia
Arquivos Brasileiros de Cirurgia Digestiva
Brazilian Journal of Craniomaxilofacial Surgery
Arquivos Brasileiros de Cirurgia Digestiva
Acta Ortopedica Brasileira
São Paulo Medical Journal
Brazilian Journal of Otorhinolaryngology
Jornal de Pediatria
Revista da Sociedade Brasileira de Cancerologia
Revista Brasileira de Reumatologia
Arquivos de Gastroenterologia
Dementia & Neuropsychologia
Revista Brasileira de Cirurgia Cabeça e Pescoço
Urologia Contemporânea
Revista Brasileira de Cirurgia Cardiovascular
Revista Medicina de Reabilitação
Brazilian Oral Research
Acta Ortopedica Brasileira
Arquivos Brasileiros de Oftalmologia
Jornal Vascular Brasileiro
Revista Brasileira de Medicina do Trabalho
